# Novel nano-pomegranates based on astragalus polysaccharides for targeting ERα-positive breast cancer and multidrug resistance

**DOI:** 10.1080/10717544.2020.1754529

**Published:** 2020-04-20

**Authors:** Bingjie Wang, Chunjing Guo, Yanhui Liu, Guangting Han, Yi Li, Yanchun Zhang, Haiyu Xu, Daquan Chen

**Affiliations:** a School of Pharmacy, Key Laboratory of Molecular Pharmacology and Drug Evaluation (Yantai University), Ministry of Education, Collaborative Innovation Center of Advanced Drug Delivery System and Biotech Drugs in Universities of Shandong, Yantai University, Yantai, P.R. China;; b State Key Laboratory of Bio-Fibers and Eco-Textiles, Qingdao University, Qingdao, P. R. China;; c School of Pharmacy, Anhui University of Chinese Medicine, Hefei, China;; d Institute of Chinese Materia Medica, China Academy of Chinese Medical Sciences, Beijing, P. R. China

**Keywords:** Astragalus polysaccharides, targeting ERα-positive breast cancer, multidrug resistance, nano-pomegranates

## Abstract

Chemotherapy is an important method for treating breast cancer. However, multidrug resistance is one of the major challenges in breast cancer chemotherapy. There is an urgent need to develop novel, effective antitumor strategies that will perfect existing therapeutic regimens. In this study, the double-targeted nanocarrier, Quercetin-3’3-dithiodipropionic acid-Astragalus polysaccharides-Folic acid (QDAF), was successfully synthesized and self-assembled into a neoteric nano-targeted delivery strategy, named nano-pomegranates, and which were utilized to effectively inhibit multidrug resistance in estrogen receptor α (ERα)-positive breast tumor. The outstanding abilities of nano-pomegranates to release the drug in a reducing environment was determined by in vitro release assay. The cellular studies in MCF-7 cells were examined that nano-pomegranates have remarkable efficiencies of enhancing cellular uptake, inhibition and necrosis and apoptosis. *In vivo* antitumor experiments showed that nano-pomegranates have better anti-tumor effects and lower systemic toxicity than free Cur. In conclusion, nano-pomegranates have great potential in anti-breast cancer treatment.

## 1. Introduction

According to ‘Global Cancer Statistics 2018’ (Bray et al., 2018), in 2018, there were approximately 2.1 million new breast cancer cases, and these accounted for almost 1 in 4 tumor cases in women. Consequently, breast cancer is the most common cancer in women. ‘Cancer Statistics, 2019’ (Siegel et al., 2019) estimated that breast cancer would account for 30% of new cancer cases in the United States in 2019. The incidence of breast cancer is constantly rising, and the treatment of this disease has faced severe challenges.

Breast cancer treatment is a painful, complicated, and long-lasting process (Arruebo et al., 2011; Pilleron et al., 2019). Simple therapies, such as surgery, chemotherapy, radiotherapy, endocrine therapy, were not able to completely extirpate or destroy tumors. Chemotherapy is an indispensable therapeutic means for all patients with cancer (Malorni et al., 2017). However, non-selectivity could easily lead to later accumulation of chemotherapeutic drugs at the tumor sites and higher systemic toxicity, and the occurrence of drug resistance (Namee & O'Driscol, 2018; Nikolaou et al., 2018; Aleksakhina et al., 2019; Ji et al., 2019). The application of nano-targeting technology in the field of medicine may resolve these issues (Tran et al., 2016; Tang et al., 2017; Liu et al., 2008; Reshma et al., 2019; Terlizzi et al., 2019). First, the nano-targeting delivery system can improve the solubility of insoluble drugs and avoid the use of a lot of surfactants. It may also achieve sustained or controlled release effects, which may reduce the relative dosage of drugs and increase their safety. In addition, surface modification can be performed to nanomaterials to allow the development of a multifunctional drug delivery system, which improve the targeting efficacies and reduced adverse reactions (Chen et al., 2017a, 2017b; Dong et al., 2018; Wang et al., 2018a, 2018b, 2018c, 2019a). Some nanomaterials with special properties, such as optics, can be used in the minimally invasive treatment of tumors (Bouramtane et al., 2019; Jia et al., 2019; Qi et al., 2019; Sun et al., 2019; Wang et al., 2019b; Yu et al., 2019; Zhao et al., 2019).

Of the different nano-delivery systems constructed, micelles are extraordinary and magnetic, owing to their unique properties. Many researchers have used micelles to construct nano-delivery systems (Greish et al., 2018; Yotsumoto et al., 2018; Du et al., 2019; Mu et al., 2019; Song et al., 2019). First, micelles are formed by the self-assembly of amphiphilic materials. If the hydrophobicity of their constituent materials changes under strong external conditions, the micelles disintegrate and the drugs are rapidly released from the micelles. Second, the micelles have a large hydrophobic cavity structure, which allows the loading of many drugs. Third, the outside of the micelles can be easily modified with many target ligands or monoclonal antibody molecules, greatly improving the targeting of micelles (Yu et al., 2018). In our study, micelles were selected to deliver curcumin (Cur) to achieve anti-breast cancer effects.

Cur, a traditional Chinese medicine active monomer extracted from turmeric, has been reported to exert many activities, such as anti-inflammatory, antibacterial, antioxidant, and antitumor effects (Abdallah et al., 2018; Barati et al., 2019; Kundur et al., 2019). Furthermore, compared with chemotherapy drugs, Chinese herbal extracts offer the major advantage of lower toxicity. In this study, Cur was selected as a model drug for the study of its anti-breast cancer effects.

Polysaccharides, composed of many sugars formed *via* different glycosidic bonds, are also significant active ingredients in traditional Chinese medicine. Traditional Chinese medicine polysaccharides have broad pharmacological activities, including immunomodulatory, anti-inflammatory, antivirus, and antioxidation effects (Zhang et al., 2017; Li et al., 2019; Xu et al., 2019b). In this work, the comparison of a variety of traditional Chinese medicine polysaccharides, Astragalus polysaccharides (APS), well-known for their immunoregulation effects, were chosen as a new excipient to assist the antitumor effects of Cur.

Quercetin (Que) is a common flavonoid, abundant in vegetables, fruits, and tea. Que has many physiological activities, such as antioxidant, antitumor, and anti-inflammatory effects (Houghton et al., 2018; Ma et al., 2018; Maurya & Vinayak, 2019; Mrkus et al., 2019). However, poor solubility and low bioavailability have greatly limited the application of Que. In our study, the hydrophobicity of Que was used to modify APS to form novel amphiphilic nano-carriers. In addition, 3,3′-dithiodipropionic acid (DA) was selected as a ligand to bind Que and APS. In addition, the disulfide bond (S-S) of DA may be rapidly degraded by high concentrations of glutathione (such as that in the tumor microenvironment, TME) (Aluri et al., 2009). Folic acid (FA) is an important ligand that specifically binds to folate receptors on the surface of the tumor cells. Thus, FA was chosen to modify APS to increase the targeting selectivity of nano-carriers (Gomhor et al., 2018; Chen et al., 2019; Ren et al., 2019; Xu et al., 2019a; Yao et al., 2019; Zhang et al., 2019).

**Figure 1.**
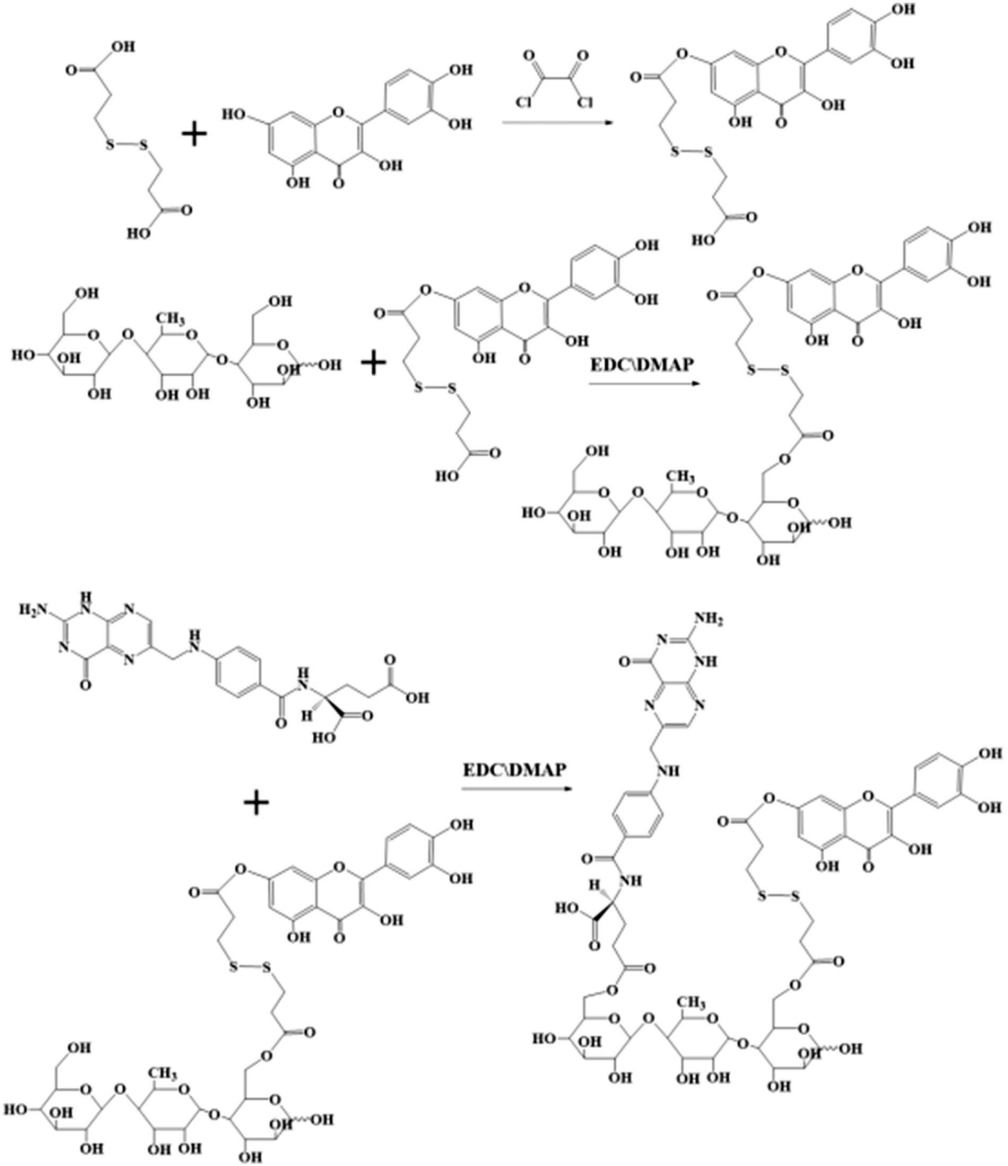
The synthesis steps of QDA and QDAF.

**Figure 2.**
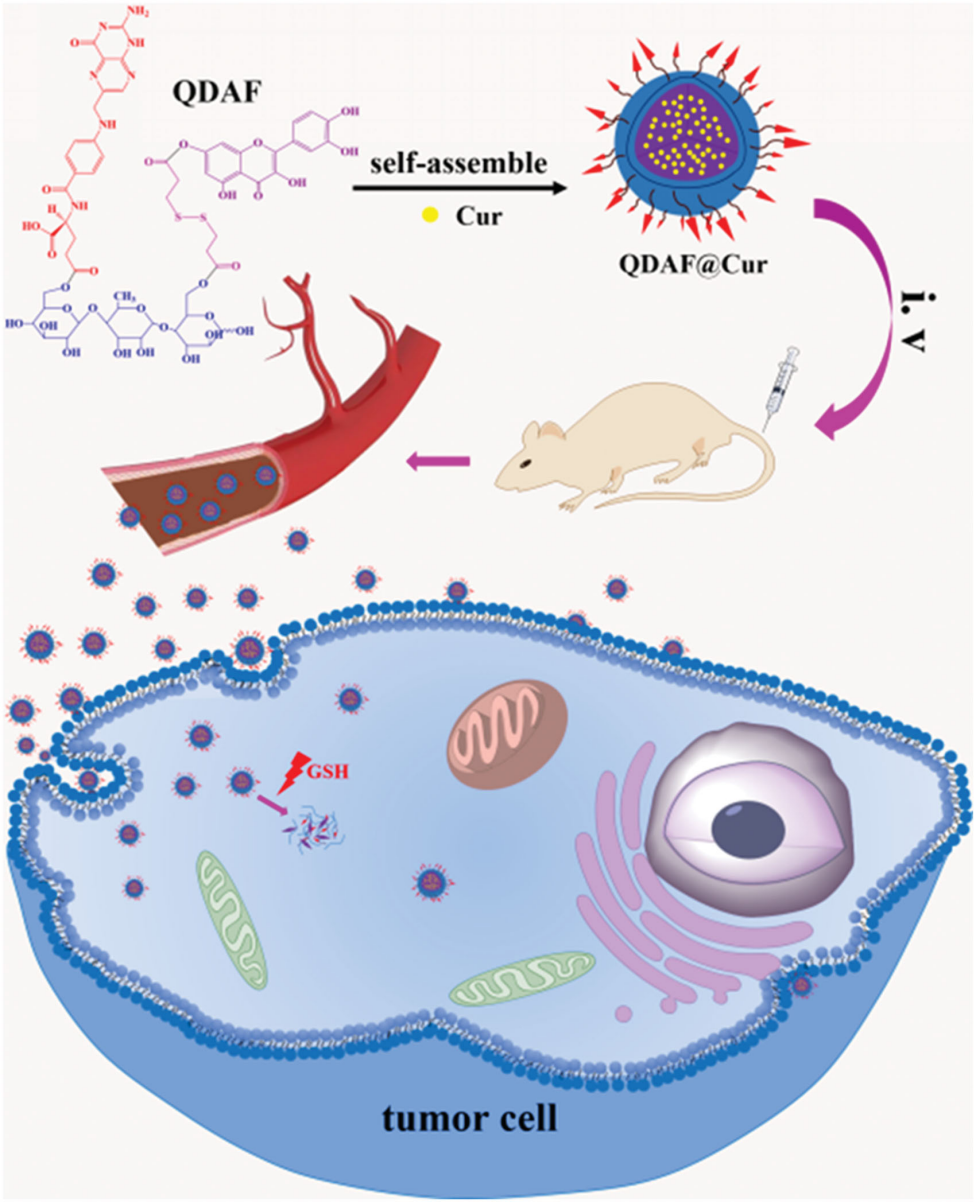
Schematic representation of QDAF self-assembly into nano-micelles and QDAF@Cur targeting to MCF-7 cells.

In this work, double-targeted nano-carriers (Quercetin-3,3′-dithiodipropionic acid-Astragalus polysaccharides-Folic acid, QDAF) with folate receptor-targeting ability and sensitivity to a reducing environment were designed and constructed (Figure 1). In addition, quercetin-3,3′-dithiodipropionic acid-Astragalus polysaccharides, QDA, was selected as the control material. As shown in Figure 2, QDAF was self-assembled into QDAF@Cur (named nano-pomegranate), parceling hydrophobic Cur and killing breast tumor cells. Nano-pomegranate is known to have a small particle size (132.2 ± 9.4 nm), spherical structure, and an appropriate surface charge (−33.71 ± 4.8 mV) to maintain a steady state in the blood circulation. *In vitro* release experiments showed that nano-pomegranate had good reduction sensitivity. Cellular assays showed that nano-pomegranate had excellent uptake ability in MCF-7 cells, inhibited growth or invasion, and promoted apoptotic and necrosis. *In vivo* real-time imaging showed that nano-pomegranate could accumulate in the tumor sites of nude mice-bearing MCF-7 cells. The *in vivo* antitumor experiments showed that nano-pomegranate exerted prominent antitumor effects and low systemic toxicity. In conclusion, nano-pomegranate was shown to be an excellent anti-breast cancer treatment platform with great potential and prospects.

## 2. Materials and methods

### 2.1. Materials

Que and APS was purchased from Yuanye Biotechnology Co. Ltd, Shanghai, China. DA, FA, formamide, tetrahydrofuran (THF), and 1-hydroxybenzotriazole hydrate (HOBT) were obtained from Aladdin Reagent Net. Dimethyl sulfoxide (DMSO) was acquired from Tianjin Fuyu Chemical Industrial Corporation. Dulbecco’s minimum essential medium (DMEM) was obtained from Saiersi Biotechnology Co. Ltd, and fetal bovine serum (FBS) was obtained from Zhejiang Tianhang Biotechnology Co. Ltd.

### 2.2. Methods

#### 2.2.1. Preparation and characterization of QDA and QDAF

The preparation of QDA and QDAF (Figure 1) was based on simple synthetic methods.

First, DA (100 mg, 0.40 mM) was dissolved in 6 mL anhydrous THF, and 100 µL oxalyl chloride was added dropwise at 0 °C. Next, the mixed solution was heated continuously at 35 °C. After 5 h, the reacted solution was subjected to rotary evaporation to remove the solvent and unreacted oxalyl chloride. Then, 1.2 equivalents of Que (123 mg, 0.48 mM) were added into the previous reaction bottle and 7 mL anhydrous tetrahydrofuran was used to dissolve the compound, which should be incubated at 45 °C for 36 h. The solvent of the final solution was removed by using a rotary evaporator. From this step, unilaterally substituted dithiodipropionic acid monoesters, Que-DA (QD), were obtained. Next, the prepared QD, 1.6 equivalents of EDCI (122.69 mg, 0.64 mM) and 1.2 equivalents of HOBT (64.86 mg, 0.48 mM) were dissolved in 3.5 mL DMSO for 3 h at 42 °C to activate the COOH groups of DA. APS (200 mg) was completely dissolved in 3.5 mL DMSO and transferred to the activated DA solution. The mixed solution was incubated at 52 °C for 48 h, and DMSO was removed by dialysis. Finally, QDA was produced by lyophilization.

FA (88.28 mg), 1.5 equivalent EDCIs of (52.82 mg, 0.3 mM), and 1.2 equivalents of HOBT (32.43 mg, 0.24 mM) were well dissolved in 4 mL DMSO by the application of heat and ultrasound. Then, the solution was stirred at 42 °C. After 4 h, 160 mg of prepared QDA was dissolved in 4 mL DMSO and added into the front of the activated solution. After the reaction was performed for 48 h, QDAF was obtained by dialysis and lyophilization, sequentially.

The prepared QDA and QDAF were verified by ^1^H-NMR spectroscopy (Chen et al., 2017b; Wang et al., 2018a, 2018b).

#### 2.2.2. Preparation of nano-pomegranates

The dialysis method was selected to prepare nano-pomegranates based on our previous experiments (Dong et al., 2018; Wang et al., 2018c). First, QDAF (10 mg) was dissolved in a 1:1 mixture of DMSO and formamide (1:1, v/v) by the application of heat and ultrasound. Second, 1.5 mg of Cur was dissolved with formamide and transferred to the QDAF solution. Third, the mixture was evenly mixed by ultrasound. Next, the mixture was transferred to a dialysis bag (2000 DA) for 24 h to remove the DMSO and formamide. Finally, the nano-pomegranates were prepared by centrifugation and filtration (0.80 µm).

#### 2.2.3. Characterization of nano-pomegranates

The mean diameters, polydispersity index (PDI), and zeta potential of QDA@Cur and QDAF@Cur were measured by Delsa Nano C (Beckman coulter, USA). The transmission electron microscope (TEM, JEM-1400 Plus) was applied to determine the morphology of QDA@Cur and QDAF@Cur. HPLC (Agilent 1260 GB12C, USA) was employed to examine the drug encapsulation efficiency (EE %) and drug loading (DL %) (Yu et al., 2018).

In addition, the stability of QDA@Cur and QDAF@Cur was investigated. The fresh anticoagulated rat blood was collected and centrifuged to obtain rat plasma. Then, 0.3 mL of prepared QDA@Cur or QDAF@Cur was diluted with 2.7 mL of the collected rat plasma to finish the plasma stability test. Diluted QDA@Cur or QDAF@Cur was divided into six groups (*n* = 3) and were then placed in an oscillating water bath (37 °C, 100 rpm). After different times (1 h, 2 h, 4 h, 8 h, 12 h, and 24 h), the size of QDA@Cur and QDAF@Cur was measured and analyzed. The stability of QDA@Cur and QDAF@Cur was evaluated in rat plasma.

#### 2.2.4. *In vitro* release study of nano-pomegranates

The *in vitro* studies of Cur release were performed by using dialysis. Phosphate buffer solution (PBS, pH = 7.4; 45 mL) was used to simulate the body fluid environment and 0.5% Tween-80 was added into PBS to improve the solubility of Cur. Condensed QDA@Cur or QDAF@Cur (1 mL) were each added to a dialysis bag (2000 Da) and immersed in PBS to examine the following release behaviors. At the specified time points, the exchange between 1 mL of fresh release medium and 1 mL of the sample medium was performed. The release of Cur was measured at 425 nm by using HPLC.

#### 2.2.5. Cell culture

In this study, MCF-7 cells, which have abundant folate receptors distributed on the cell surface, were used to evaluate the cellular levels of QDA@Cur and QDAF@Cur. MCF-7 cells were maintained in DMEM supplemented with 10% FBS. In addition, FBS supplemented with 10% DMSO was used to preserve cells at −80 °C.

#### 2.2.6. Biocompatibility evaluation of QDAF

The MTT method was used for the study of the biocompatibility of QDAF. The MCF-7 cells were seeded in 96-well plates for 12 h. Bactericidal QDAF was dissolved at 500, 200, 100, 50, and 20 µg/mL in DMEM, added to 96-well plates, and cultured for 24 or 48 h. MTT (20 µL) was added into the medium and the cells were cultured in the dark. After incubation for 4 h, the medium was removed and 200 µL DMSO was added into each well. Finally, the absorbance of cell plate was read at 570 nm by using a microplate reader.

#### 2.2.7. *In vitro* cytotoxicity study

The MTT assay was used to study the cytotoxicity of QDA@Cur and QDAF@Cur. First, MCF-7 cells were seeded in 96-well plates for 12 h. Then, the cells were treated with free Cur, QDA@Cur, and QDAF@Cur (Cur concentrations: 20, 10, 5, 2, 1, 0.5 µg/mL) for 24 h or 48 h. Then, 20 µL MTT was added into the medium and the cells were cultured in dark. After 4 h, the medium was replaced by 200 µL DMSO. Finally, the absorbance of each well was detected by using a microplate reader, and the cell viability was calculated from a previously reported formula (Wang et al., 2018b).

#### 2.2.8. Cellular uptake study

In this experiment, a fluorescence inverted microscope was used to compare the cellular uptake of the different nano-micelles (NMs) in MCF-7 cells.

First, the concentration dependence study was monitored. MCF-7 cells were seeded in 12-well plates and allowed to adhere for 12 h. Meanwhile, free Cur, QDA@Cur, and QDAF@Cur were prepared and diluted to the desired concentrations (Cur concentrations: 20, 10, 5, and 2.5 µg/mL). After culture, the medium was replaced by different NMs and the cells were cultured for 4 h. After the cells were washed and fixed, fluorescence imaging was performed by using an inverted fluorescence microscope.

Second, a time-dependence study was performed. The cell culture methods used were the same as for the concentration-dependence study. Free Cur, QDA@Cur, and QDAF@Cur were diluted to the necessary concentration (Cur concentration: 20 µg/mL) and added to the cells and cultured for different times (0.5 h, 1 h, 2 h, and 4 h). Subsequently, the cells were washed, fixed, and fluorescence imaging was performed by using an inverted fluorescence microscope.

#### 2.2.9. Cellular invasion assay

First, the MCF-7 cells were starved by culture in serum-free DMEM. Matrigel was placed at 4 °C to melt into a liquid, and 100 µL Matrigel was poured onto the surface of the upper chamber of the Transwell plate. The Transwell plate was kept at 37 °C until the Matrigel was completely solidified. Then, 200 µL serum-starved MCF-7 cells (5 × 105 cells/mL) were prepared in serum-free DMEM (Cur concentration: 0, 2.5, 5, 10, and 20 µg/mL) and seeded in the upper chamber. The lower chamber was filled with DMEM supplemented with 10% FBS for 24 h. Next, after washing, the cells were fixed in 4% paraformaldehyde. Then, the cells were stained with crystal violet. Finally, pictures of invaded cells were taken by using a light microscope and the number of cells in the pictures was counted.

#### 2.2.10. Cellular apoptosis and necrosis assay

In these experiments, the Apoptosis and Necrosis Assay Kit was used. First, MCF-7 cells were seeded in 12-wells. After 12 h, the next experimental procedures were conducted in accordance with the concentration-dependent cellular uptake experimental procedures until the end of dosing. Then, 5 µL propidium iodide (PI) and 5 µL Hoechst 33342 were added into the medium, which was shaken and stored at 4 °C for 30 min. Next, the cells were washed twice and the fluorescence imaging was performed by using an inverted fluorescence microscope.

#### 2.2.11. Hemolysis experiment

The hemolysis experiments were used to evaluate the safety of QDAF@Cur before use *in vivo*.

Anti-coagulant rat blood (2 mL) was placed in a conical flask containing glass beads and shaken for 10 min to remove fibrinogen. Then, approximately 10 volumes of saline were added into the conical flask and mixed. After centrifugation for 8 min at 3000 r/min, the supernatant was removed and the precipitated red blood cells were washed three times with saline. The obtained red blood cells were mixed with saline to 2% (v: v).

To six tubes, 2% red blood cell suspension, saline, and QDAF@Cur were added as shown in Table 1, mixed, and placed in an incubator (37 °C) for 2 h. Finally, all tubes were collected, centrifuged, and the hemolysis of the supernatant was observed.

**Table 1.** Samples for hemolysis and agglomeration testing of QDAF@Cur by *in vitro* naked-eye observation method.

Group	1	2	3	4	5	6
2% red blood cell suspension (ml)	2.5	2.5	2.5	2.5	2.5	2.5
saline (ml)	2	2.1	2.2	2.3	2.4	2.5
QDAF@Cur (ml)	0.5	0.4	0.3	0.2	0.1	0

#### 2.2.12. *In vivo* real-time imaging

*In vivo* fluorescence imaging is an important method used for the evaluation of anticancer research.

In this study, DiR was selected as the fluorescent probe that was loaded in the various NMs for the visualization of NMs in MCF-7 cells-bearing nude mice. The nude mice bearing MCF-7 cells were injected with free DiR, QDA@DiR, or QDAF@DiR via the tail vein. The mice were photographed at set times by using an IVIS Spectrum Pre-Clinical In Vivo Imaging system (Perkin Elmer, Waltham, MA, USA).

#### 2.2.13. *In vivo* pharmacodynamic evaluation and histological analysis

To more clearly recognize the therapeutic effects and the damage to the main organs induced by free Cur, QDA@Cur, and QDAF@Cur, *in vivo* anti-tumor therapeutic efficacy evaluations were conducted. First, nude mice bearing MCF-7 cells were randomly divided into four groups (*n* = 5). Then, the saline, free Cur, QDA@Cur, and QDAF@Cur were injected *via* the tail vein every other day. Throughout the treatment process, some indices of mice were recorded before each administration, such as the body weight, tumor length, and tumor width. At the end of the administration period, the mice were humanely sacrificed. The major organs and tumor were collected and immersed in 4% paraformaldehyde. After dehydration and embedding, the organs and tumor were sliced and the sections were stained with hematoxylin and eosin (HE). Finally, the stained sections were observed by using a light microscope.

#### 2.2.14. Statistical analyses

All data were analyzed by using Student’s *t*-test and ANOVA. The quantitative data were presented as the mean ± standard deviation (SD).

## 3. Results

### 3.1. Synthesis of QDA and QDAF

**Figure 3.**
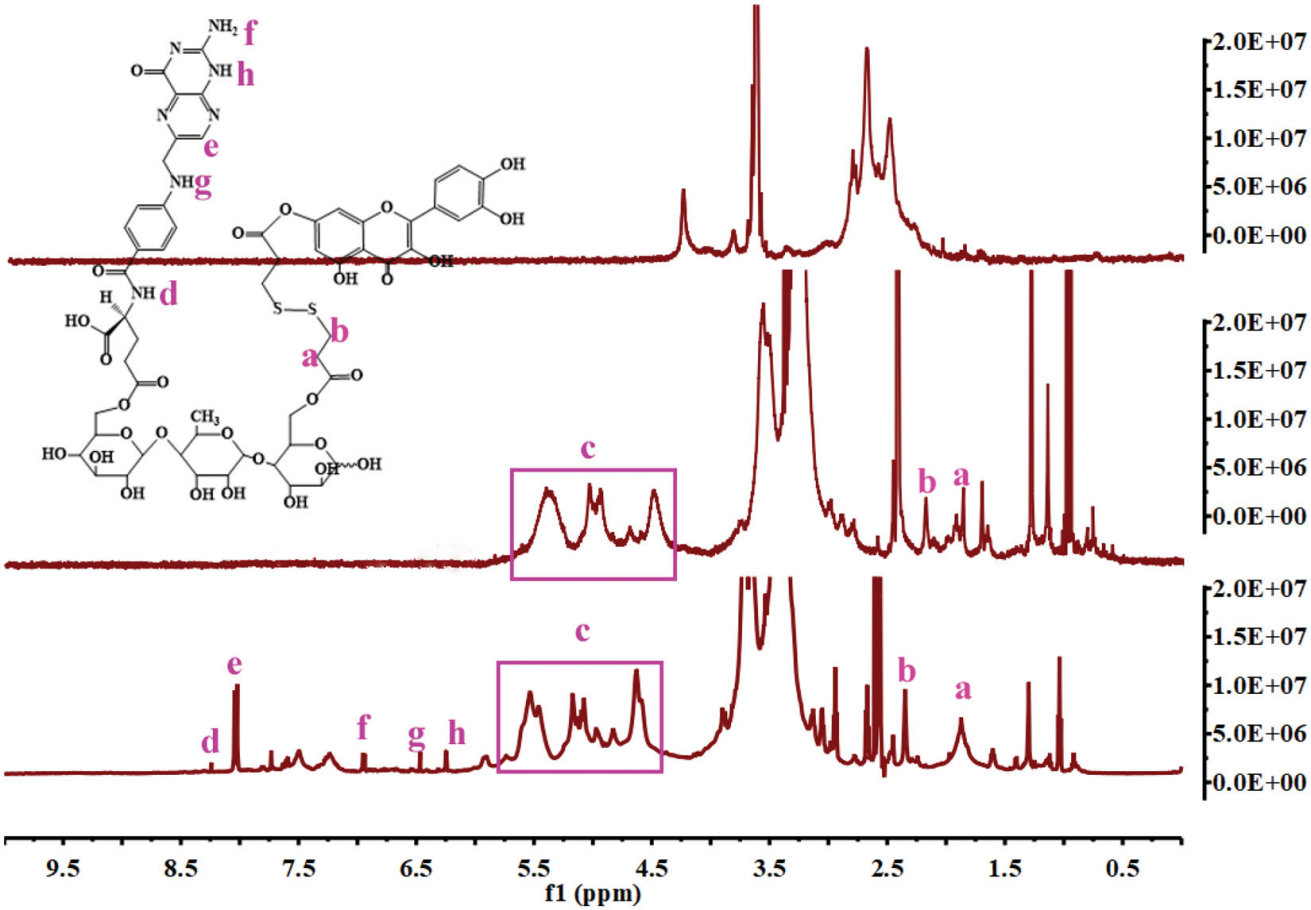
The ^1^H-NMR spectra of QDA and QDAF.

QDA and QDAF were synthesized and their structures were confirmed by using ^1^H-NMR (Figure 3). Clear new chemical shifts were present at 2.4 ppm (b) and 1.8 ppm (a) and assigned to DA (COO-CH_2_-CH_2_-S), and the chemical shifts at 4.5–6 ppm (c) was the peak of Que (benzene ring). The chemical shifts at d (8.25 ppm), e (8.03 ppm), f (6.98 ppm), g (6.50 ppm), h (6.25 ppm) were confirmed belonging to FA. All results confirmed that QDA and QDAF were successfully synthesized.

### 3.2. Characterization of nano-pomegranates

**Figure 4.**
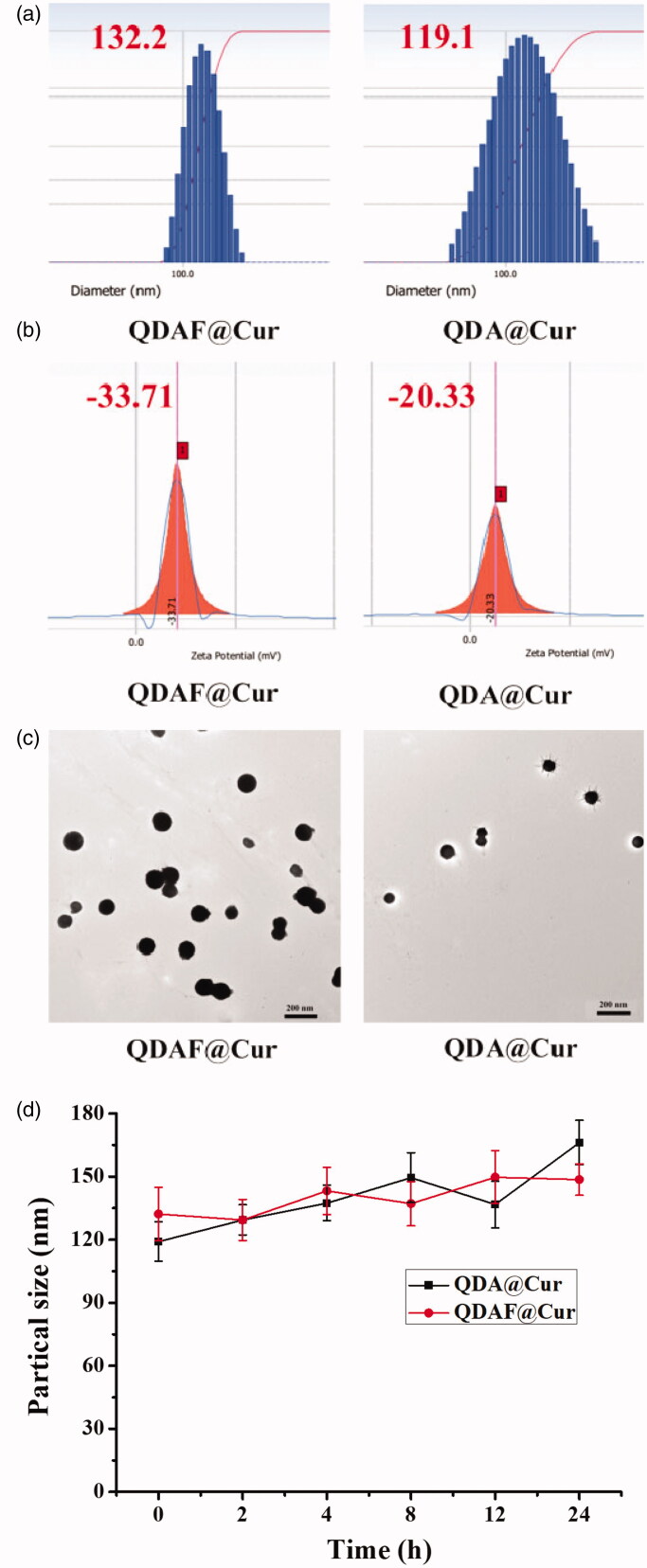
The particle size (a), zeta potential (b), TEM morphology, (c) and stability (d) of QDA@Cur and QDAF@Cur (scale bar =200 nm).

The average diameter, zeta potential, and TEM morphology of QDA@Cur and QDAF@Cur are shown in Figure 4. As shown in Figure 4(a), QDAF@Cur was slightly larger than QDA@Cur, which may be attributable to the hydrophobic modification of QDA by FA. Compared with QDA@Cur, QDAF@Cur had slightly larger negative charges (Figure 4(b)) conferring greater stability in the blood-circulation, avoiding self-aggregation and binding to plasma proteins. The TEM morphology (Figure 4(c)) showed that both QDA@Cur and QDAF@Cur were spherical in structure. The detailed data on the particle size, PI, zeta potential, EE, and DL of QDA@Cur and QDAF@Cur are presented in Table 2.

**Table 2.** The particle size, PI, zeta potential, EE, and DL of QDA@Cur and QDAF@Cur.

Group	particle size (nm)	P. I.	zeta potential (mV)	EE (%)	DL (%)
QDA@Cur	119.1 ± 12.7	0.126	−20.33 ± 3.6	43.52 ± 7.2	6.12 ± 1.01
QDAF@Cur	132.2 ± 9.4	0.134	−33.71 ± 4.8	51.31 ± 10.6	7.14 ± 1.48

As shown in Figure 4(d), there was a small fluctuation in the particle sizes of QDA@Cur and QDAF@Cur. That is, QDA@Cur and QDAF@Cur maintained a stable morphology with no obvious aggregation.

### 3.3. *In vitro* release study of nano-pomegranates

**Figure 5.**
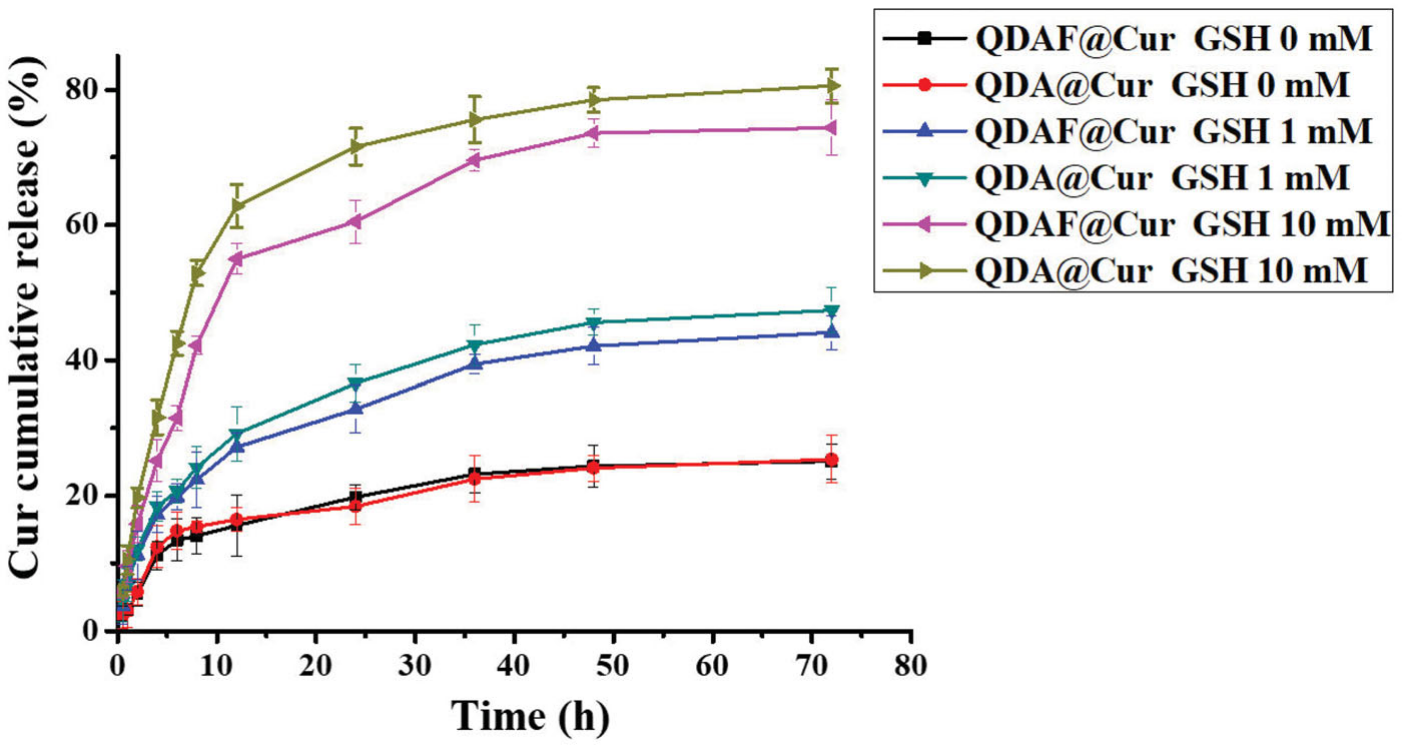
*In vitro* release of Cur from QDA@Cur and QDAF@Cur in medium containing 0, 1, and 10 mM of GSH.

*In vitro* drug release behavior was and is presented in Figure 5. Cur was released quickly from QDA@Cur or QDAF@Cur as the concentration of GSH in the release medium was increased. In particular, when the concentration of GSH reached 10 mM, the cumulative release of Cur was approximately 80% from QDA@Cur and approximately 75% from QDAF@Cur, which was slightly less than QDA@Cur. This was attributed to the presence of FA (a hydrophobic structure) in QDAF, which retarded the degradation in higher GSH concentrations. In summary, both QDA@Cur and QDAF@Cur showed noticeable sensitivity to the reducing environment, which promoted rapid Cur release rapidly in the tumor site in the presence of a high concentration of GSH, to concentrations required to be kill the cancer cells.

### 3.4. Biocompatibility evaluation of QDAF

**Figure 6.**
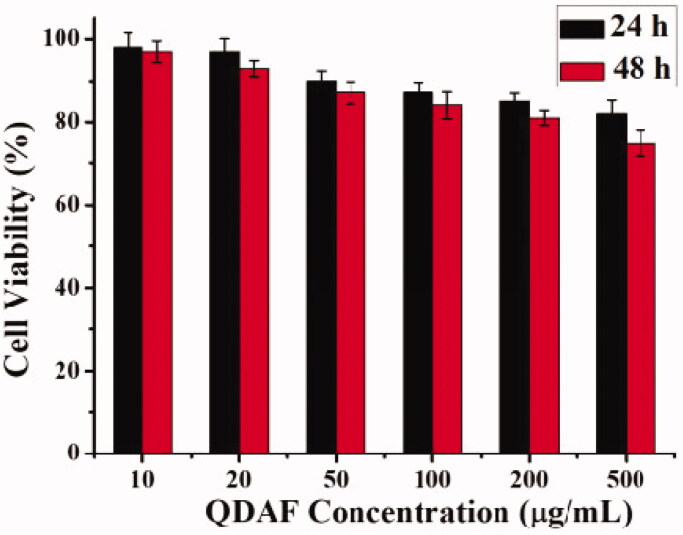
Biocompatibility of QDAF.

The results of the QDAF-biocompatibility are shown in Figure 6. The cell viability was approximately 83% at 24 h and about 78% at 48 h, which confirmed that QDAF had lower cytotoxicity to MCF-7 cells and was more biocompatible.

### 3.5. *In vitro* cytotoxicity study

**Figure 7.**
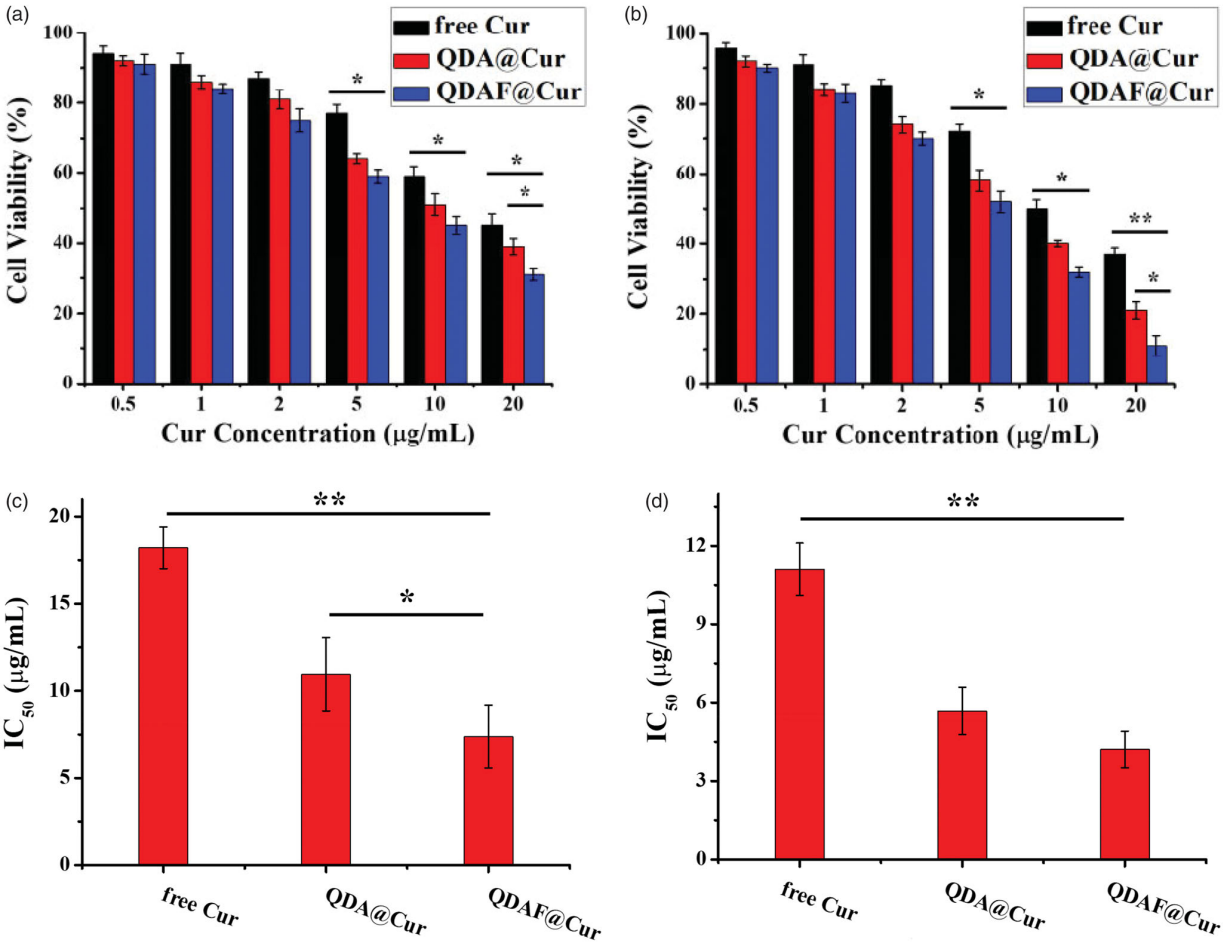
*In vitro* cytotoxicity of free Cur, QDA@Cur, and QDAF@Cur in MCF-7 cells after treatment for 24 h (a) and 48 h (b). The IC_50_ of free Cur, QDA@Cur, QDAF@Cur after administration for 24 h (c) and 48 h (d).

The cytotoxicity of free Cur, QDA@Cur, and QDAF@Cur was tested in MCF-7 cells. The MTT method was used to evaluate the cell viability (Figure 7).

Figure 7(a,b) shows the cytotoxicity of free Cur, QDA@Cur, and QDAF@Cur to MCF-7 cells after administration for 24 h and 48 h. In Figure 7(a,b), the cell viability decrease with an increase in the concentration of Cur. QDAF@Cur exhibited more stronger cytotoxicity than free Cur and QDA@Cur at equivalent Cur concentrations. As shown in Figure 7(a), 20 µg/mL free Cur, QDA@Cur, and QDAF@Cur reduced cell viability by up to approximately 45%, 39%, and 31% after 24 h, respectively. In Figure 7(b), 20 µg/mL free Cur, QDA@Cur, and QDAF@Cur reduced the cell viability by up to approximately 37%, 21%, and 11% after 48 h. In addition, the half maximal inhibitory concentration (IC_50_) value is shown in Figure 7(c,d). The IC_50_ values of free Cur, QDA@Cur, and QDAF@Cur were 18.21, 10.95, and 7.38 µg/mL, respectively, after administration for 24 h (Figure 7(c)). In addition, the IC_50_ values of free Cur, QDA@Cur, and QDAF@Cur were 11.11, 5.68, and 4.21 µg/mL, respectively, after administration for 48 h (Figure 7(d)).

The higher toxicity of QDAF@Cur was suggested to occur because of the presence of FA, which should have a high affinity for MCF-7 cells because of the exclusively binding between FA and FA receptors. These results confirmed that the novel QDAF@Cur was a potential nano-delivery system for breast cancer treatment.

### 3.6. Cellular uptake study

**Figure 8.**
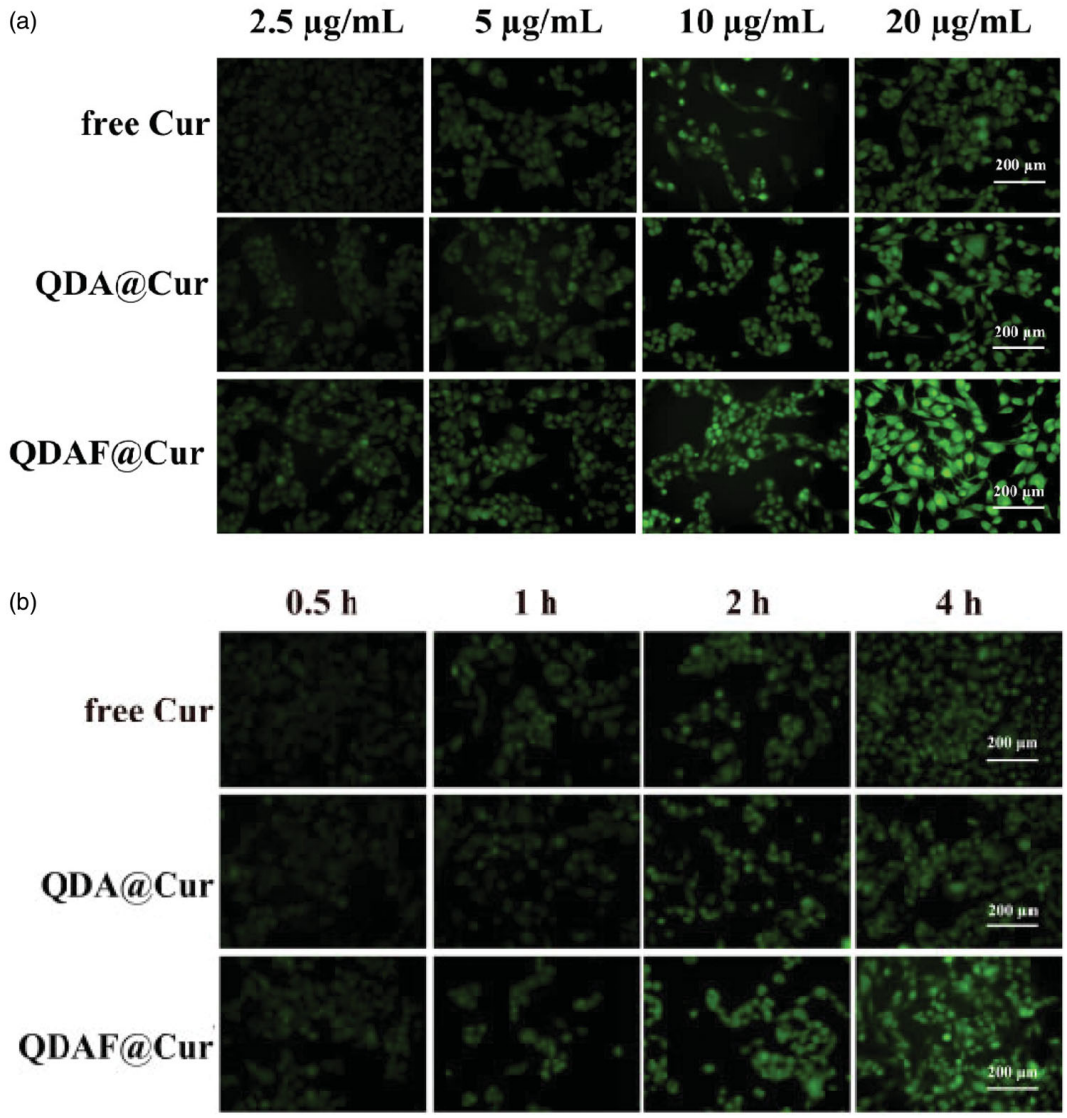
The cellular uptake of free cur, QDA@Cur, and QDAF@Cur (scale bar =200 µm).

The intensity of fluorescence was representative of the Cur uptake by MCF-7 cells; stronger fluorescence indicated greater Cur uptake. As shown in Figure 8(a), over the same administration time, the fluorescence intensity was significantly increased with an increase in the Cur concentration, which indicated that the internalization of free Cur, QDA@Cur, and QDAF@Cur was a concentration-dependent process. The same phenomenon was apparent from Figure 8(b). At an equal Cur concentration, the fluorescence intensity was significantly increased with an increase in treatment time, which also confirmed that the uptake was a time-dependent process. In addition, when the administration time and Cur concentration was the same, the cellular uptake effects of these formulations were ranked in following order: QDAF@Cur > QDA@Cur > free Cur; this order was attributed to the specific ligand-mediated targeted delivery of QDAF. Overall, the above results confirmed that QDAF@Cur, with better tumor-targeted delivery efficiency, showed good prospects for an anti-breast cancer treatment.

### 3.7. Cellular invasion assay

**Figure 9.**
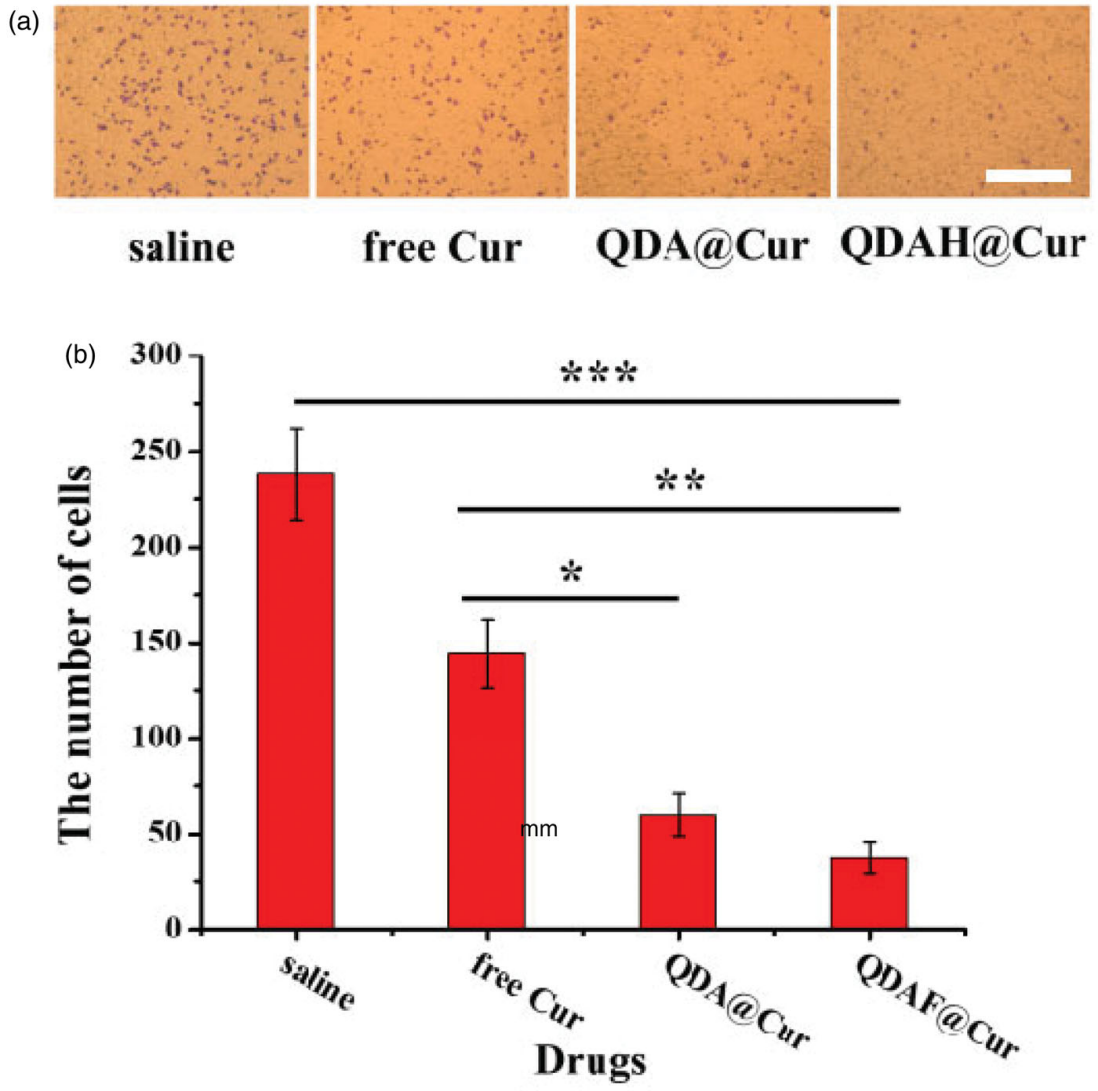
Cell mobility and invasion were examined by the Matrigel invasion assay (scale bar =500 µm).

To clarify whether QDAF@Cur was able to inhibit the invasion ability of MCF-7 cells, Matrigel invasion experiments were performed. As shown in Figure 9(a), when MCF-7 cells were pretreated with the various formulations, the number of invading cells was clearly suppressed. The data are shown in Figure 9(b), providing a more intuitive reflection of the inhibitory effects of Cur on cell invasion. In addition, it can be clearly seen that QDAF@Cur had stronger anti-invasive activity than free Cur and QDA@Cur, which was consistent with the previous cellular uptake and cytotoxicity results. The above experimental results showed that QDAF@Cur had stronger inhibitory effects on tumor cells and was a promising formulation for targeted therapy of tumors.

### 3.8. Cellular apoptosis and necrosis assay

**Figure 10.**
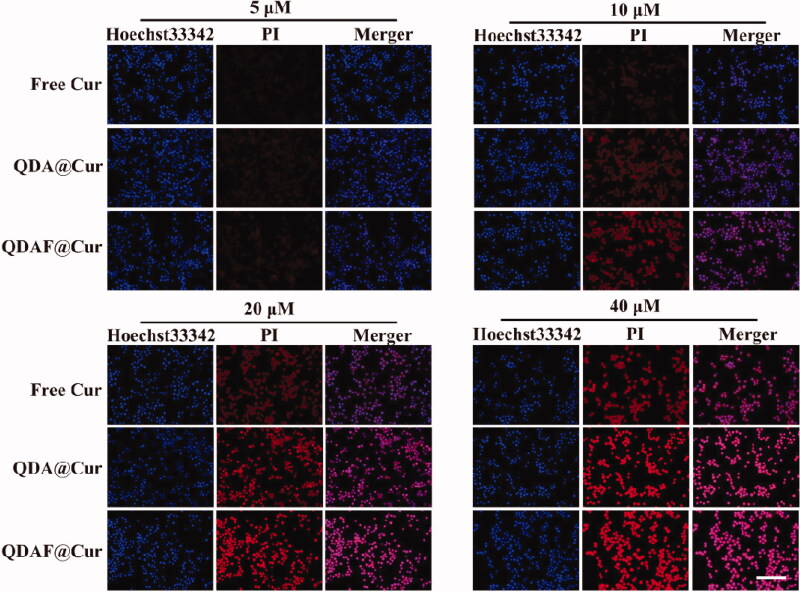
Cellular apoptosis and necrosis were examined by the PI and Hoechst 33342 staining assay (scale bar = 200 µm).

In this experiment, PI and Hoechst 33342 staining were used to dye the nucleus, although there was a difference in their staining methods. Hoechst 33342 is able to penetrate the cell membrane easily and stain the nucleus of all cells. However, it is difficult for PI to penetrate the intact cell membrane. In short, PI can only penetrate the cell membrane, and stain the nucleus, when the cell is dead and damaged. The results of the cellular apoptosis and necrosis assay are shown in Figure 10. As the concentration of Cur increased, the number of necrotic or apoptotic MCF-7 cells was also gradually increased. Especially, at a Cur concentration of 20 µM, a high proportion of MCF-7 cells showed necrosis and death. It was confirmed that Cur could induce and promote the necrosis and apoptosis of MCF-7 cells, and that this process was concentration-dependent. In addition, it was apparent that Cur-mediated cellular necrosis and apoptosis were also time dependent. This was thought to occur because MCF-7 cellular uptake and cytotoxicity were concentration- and time-dependent. Interestingly, the proportion of apoptosis and necrosis induced by the various preparations was different at the same Cur concentration. The effects of QDAF@Cur were stronger than those of QDA@Cur and free Cur. This was attributable to the introduction of FA in QDAF@Cur, which resulted in greater uptake of QDAF@Cur to by MCF-7 cells owing to the specific binding between FA and FA receptors. In summary, QDAF@Cur showed a strong ability to promote the apoptosis and necrosis of MCF-7 cells.

### 3.9. Hemolysis experiments

Hemolysis experiments were necessary before the use of QDAF@Cur in animal experiments. No obvious hemolysis and aggregation were observable by the naked eyes in all test tubes, which confirmed that the prepared QDAF@Cur was suitable for used animal experiments.

### 3.10. *In vivo* real-time imaging

**Figure 11.**
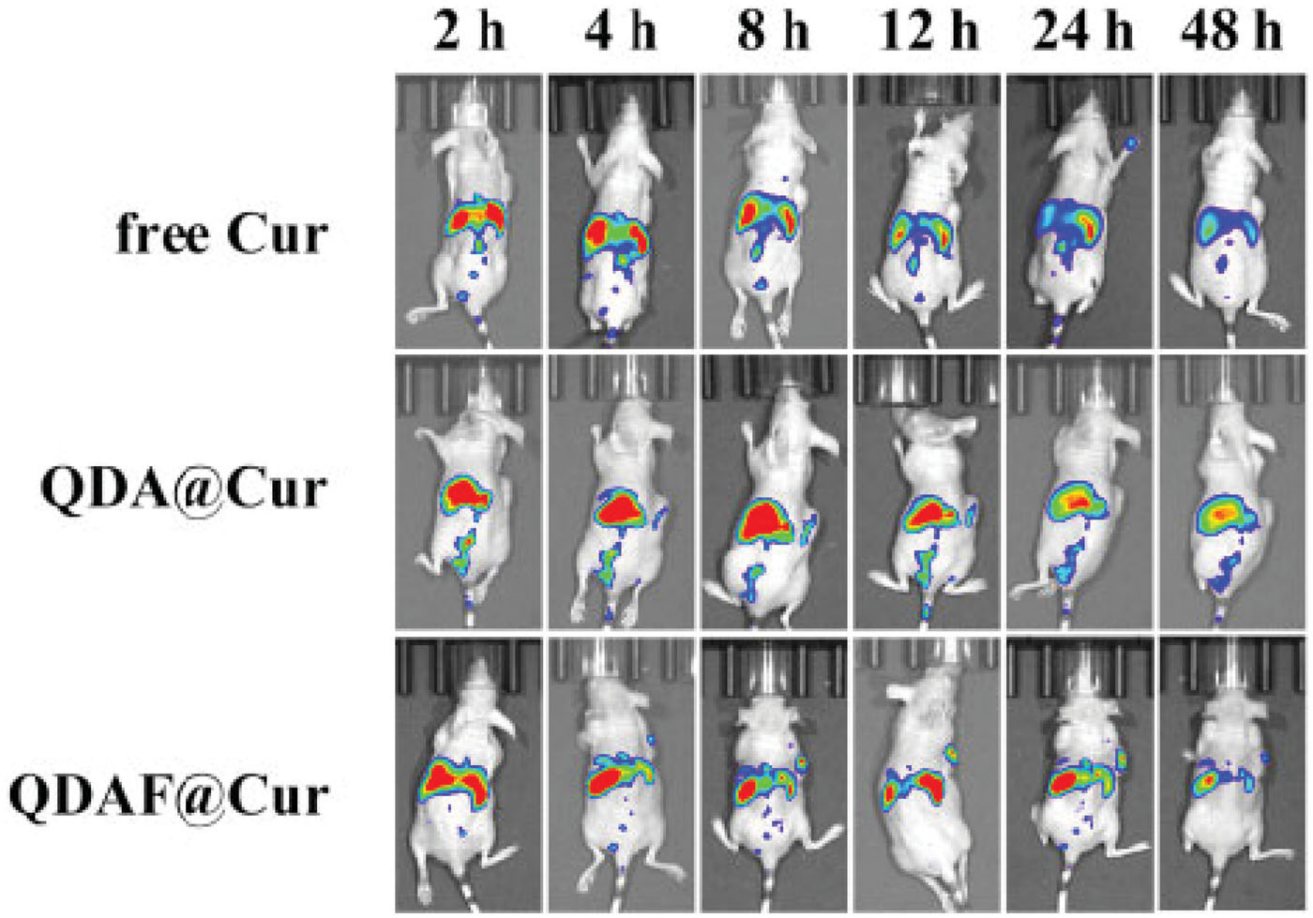
The *in vivo* fluorescence images of free DiR, QDA@DiR, and QDAF@DiR.

The *in vivo* fluorescence images of free DiR, QDA@DiR, and QDAF@DiR are shown in Figure 11. Fluorescence was detected at the tumor site at 2 h after injection, and the fluorescence intensity increased with an increase in time. As we could see from Figure 11, the fluorescence intensity of the QDAF@DiR-treated group was stronger at the tumor compared with the free DiR- and QDA@DiR-treated groups. The duration of fluorescence at the tumor site in the QDAF@DiR-treated group was longer than that in free DiR- and QDA@DiR-treated groups; moreover, the accumulation of fluorescence at the tumor site was barely observed in the free DiR-treated group. These results indicated that QDAF@DiR was gradually enriched in tumor tissues owing to the enhanced permeability and retention (EPR) effect (Kalyane et al., 2019; Tahmasbi Rad et al., 2019; Yoshikawa et al., 2019) and the exclusive binding between FA and FA receptors. DiR also showed greater systemic distribution in the free DiR- and QDA@DiR-treated groups, and the systemic distribution in the QDAF@DiR-treated group was almost zero. This indicated that QDAF@DiR could reduce the systemic toxicity of the formulations. These results were consistent with the previous experimental results for cellular uptake and toxicity.

### 3.11. *In vivo* pharmacodynamic evaluation and histological analysis

**Figure 12.**
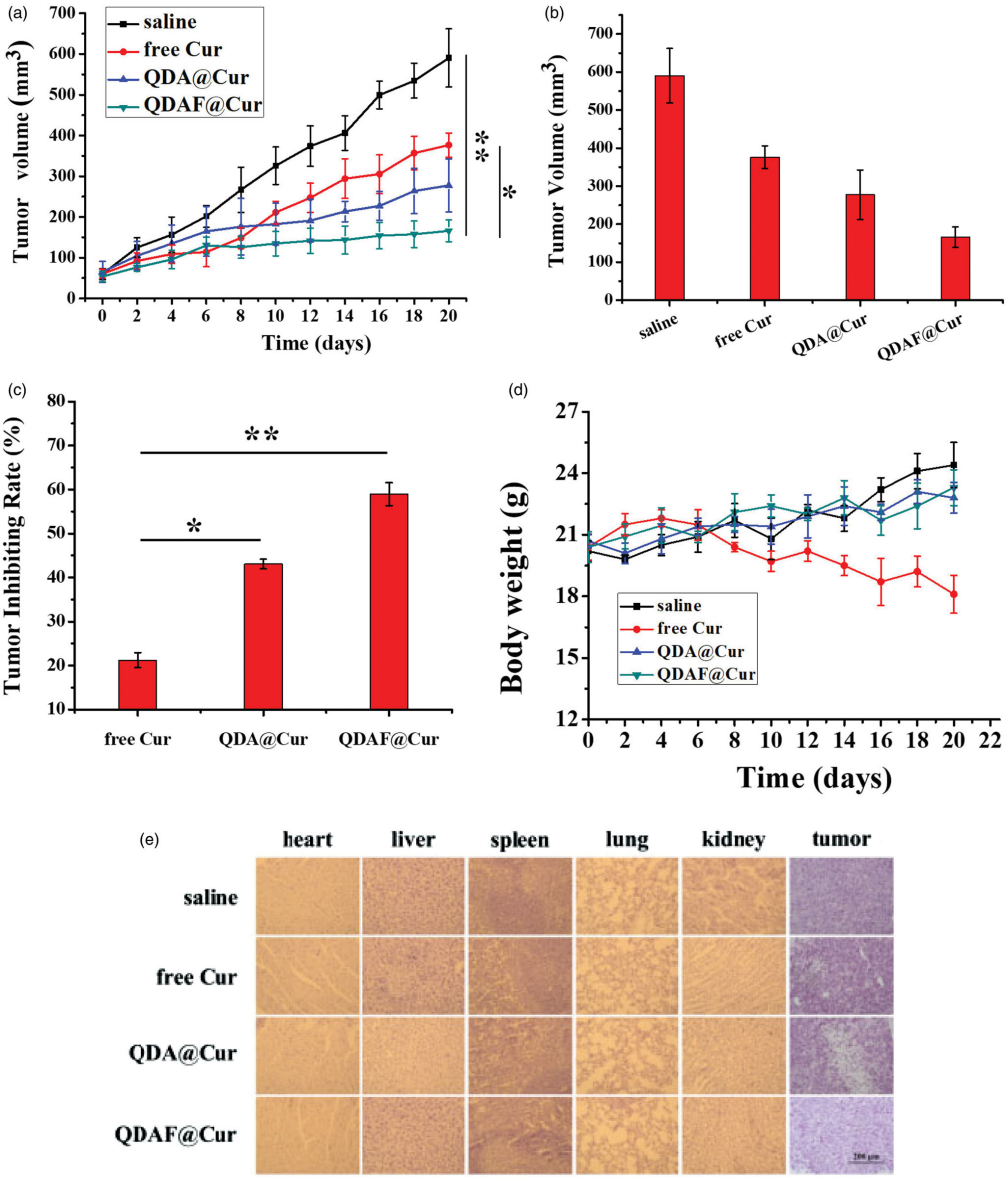
*In vivo* antitumor activity of NMs in nude mice bearing MCF-7 cells. (a) Tumor size. (b) Changes in tumor volume over time. (c) Tumor inhibition rate of various NMs. (d) Changes in the body weights of mice during treatment. (e) Histological images of tumors and major organs after different preparation processing (scale bar = 200 µm).

The anticancer effects of QDA@Cur and QDAF@Cur were further investigated in MCF-7 tumor-bearing nude mice and compared with the effects of free Cur as a control. As shown in Figure 12(a,b), all the formulation-treated groups clearly exhibited significant cancer inhibition effects compared with the saline-treated group, and the tumor-inhibiting effects of QDAF@Cur significantly outperformed those of QDA@Cur and free Cur. As shown in Figure 12(c), the tumor inhibition rate (TIR) of QDAF@Cur, QDA@Cur, and free Cur was approximately 59.94%, 43.02%, and 21.25%. Thus, it is clear that QDAF@Cur has better antitumor effects than QDA@Cur and free Cur. We speculated that the targeting ligand of FA was a crucial component of this role, greatly increasing the phagocytosis of QDAF@Cur by tumor cells. This allowed a large concentration of the loaded drug (Cur) to accumulate at the tumor site.

In addition, to evaluate the *in vivo* toxicity resulting from systemic drug delivery, the body weight of the mice was measured. No significant differences were found in the change of body weight in the four treatment groups. As shown in Figure 12(d), with the exception of mice in the free Cur-treated group, all mice exhibited appreciably increased body weight at the end of the study, which confirmed that the nano-pomegranates were able to reduce or relieve the toxicity and side effects of free Cur. The enhanced tumor selectivity and preservation were the main contributors to the clear reduction in the systemic toxicity of the nano-pomegranates. For a further examination of the toxic side effects and antitumor effects of each preparation, the tumor tissues and the vital organs of nude mice were collected and stained with HE. These results are shown in Figure 12(e), and further confirmed the above results. The clear anticancer efficacy and almost total lack of organ damage induced by QDAF@Cur and QDA@Cur were well demonstrated by HE staining. Contrary, liver injury was found in the free Cur-treated group. What’s more, obvious cellular shrinkage appeared in the tumor tissue of the QDAF@Cur, QDA@Cur and free Cur treated groups, especially QDAF@Cur treated group. In conclusion, QDAF@Cur with better anti-tumor than QDA@Cur and free Cur was a promising nano-delivery system with good tumor targeting function and lower toxicity and side effects compared with the free drug.

## 4. Discussion

It has been confirmed that Cur has anticancer effects. However, lower water solubility and distribution to the tumor locations have severely limited its widespread application. In this study, a cancer-targeting carrier material (QDAF) was designed to construct with Cur-loaded nano-pomegranates. The not only improved the water solubility of Cur, but also increased the concentration of Cur in the tumor site, which could significantly improve the tumor therapeutic effects of Cur.

In the cellular apoptosis and necrosis experiments, it was verified that QDAF@Cur induce and cellular apoptosis and necrosis. However, it was not clear at which stage of the cell division cycle this was specific to, and the in-depth mechanism was not determined. It has been reported that Cur could induce autophagy-mediated cell death through a reduction in the expression of Rictor (an mTORC2 complex protein) and Akt (Seo et al., 2018). Cur also has the potential to regulate ROS levels in canner cells, thereby controlling canner growth (Larasati et al., 2018). The specific and detailed mechanism requires further verification.

*In vivo* real-time imaging experiments revealed that DiR accumulated in the liver and spleen, which was inevitable. As particles in nano-medicine are of a small size, dozens or hundreds of nanometers, they are easily trapped by the reticuloendothelial system, resulting drug accumulation. In this study, we tried to ameliorate this defect by introducing some macromolecular polysaccharides. However, there were still some nano-pomegranates that cannot escape the phagocytosis of the reticuloendothelial system. We will continue to identify and introduce other materials that have the ability to evade the engulfment of the reticuloendothelial system, reducing the issues associated with nanoparticle treatment.

## 5. Conclusion

In this study, a cancer targeted nano-material (QDAF) was designed and successfully synthesized, which used to form the structure QDAF@Cur, which showed increased anti-breast tumor activity. This nano-targeted delivery formulation not only avoided solubility issues of Cur by loading it into the internal cavity of QDAF@Cur, but also realized the combination and application of multiple targets *via* S-S and FA.

Preliminary *in vitro* characterization confirmed that QDAF@Cur had a small particle size (132.2 ± 9.4 nm) and a suitable surface charge (-33.71 ± 4.8 mV) to confer stability in the blood circulation. TEM revealed that QDAF@Cur was spherical. *In vitro* release studies found that QDAF@Cur had good GSH-responsiveness, which allowed it to rapidly rupture and release Cur in the presence of high concentration of GSH at the TME to effectively inhibit or kill tumor tissue. The evaluation of cellular levels revealed that QDAF@Cur was taken up by MCF-7 cells, and that this uptake behavior occurred in a time-dependent and concentration-dependent manner. In addition, QDAF@Cur significantly inhibited the growth of, or killed, MCF-7 cells, as shown in the cellular cytotoxicity test, and exhibited good ability to inhibit MCF-7 cell invasion. In the apoptosis and necrosis experiments, QDAF@Cur demonstrated a strong ability to promote MCF-7 cells apoptosis and necrosis. *In vivo* real-time imaging experiments confirmed that QDAF@Cur effectively aggregated at the tumor site and ameliorated the systemic distribution of free Cur. The pharmacodynamics assessment of QDAF@Cur also revealed good antitumor potential and lower systemic toxicity.

In summary, QDAF@Cur has been shown to be a nano-drug delivery system with broad research appeal, and we believe it can provide a good research platform for future anti-breast cancer treatments.
